# Enabling one-pot Golden Gate assemblies of unprecedented complexity using data-optimized assembly design

**DOI:** 10.1371/journal.pone.0238592

**Published:** 2020-09-02

**Authors:** John M. Pryor, Vladimir Potapov, Rebecca B. Kucera, Katharina Bilotti, Eric J. Cantor, Gregory J. S. Lohman

**Affiliations:** 1 Research Department, New England Biolabs, Ipswich, Massachusetts, United States of America; 2 Applications and Product Development, New England Biolabs, Ipswich, Massachusetts, United States of America; University of Helsinki, FINLAND

## Abstract

DNA assembly is an integral part of modern synthetic biology, as intricate genetic engineering projects require robust molecular cloning workflows. Golden Gate assembly is a frequently employed DNA assembly methodology that utilizes a Type IIS restriction enzyme and a DNA ligase to generate recombinant DNA constructs from smaller DNA fragments. However, the utility of this methodology has been limited by a lack of resources to guide experimental design. For example, selection of the DNA sequences at fusion sites between fragments is based on broad assembly guidelines or pre-vetted sets of junctions, rather than being customized for a particular application or cloning project. To facilitate the design of robust assembly reactions, we developed a high-throughput DNA sequencing assay to examine reaction outcomes of Golden Gate assembly with T4 DNA ligase and the most commonly used Type IIS restriction enzymes that generate three-base and four-base overhangs. Next, we incorporated these findings into a suite of webtools that design assembly reactions using the experimental data. These webtools can be used to create customized assemblies from a target DNA sequence or a desired number of fragments. Lastly, we demonstrate how using these tools expands the limits of current assembly systems by carrying out one-pot assemblies of up to 35 DNA fragments. Full implementation of the tools developed here enables direct expansion of existing assembly standards for modular cloning systems (e.g. MoClo) as well as the formation of robust new high-fidelity standards.

## Introduction

DNA assembly methodologies are routinely used in the field of synthetic biology to generate large, complex recombinant DNA constructs from smaller fragments [1]. Golden Gate assembly is a DNA assembly methodology that has been particularly useful in these applications as it supports assembly of multiple DNA fragments in a single reaction and is amenable to automation [2–4]. Golden Gate assembly utilizes a Type IIS restriction enzyme to generate DNA fragments with compatible overhang sequences, and a DNA ligase to join the fragments together. Type IIS restriction enzymes cleave outside of their recognition sequence [5]. This feature permits assembly of DNA fragments without the need to introduce an unwanted sequence at fusion sites, enables generation of overhangs of arbitrary sequence independent of the recognition sequence, and allows the recognition sequence to be removed from the generated fragment. The ability to choose any overhang sequence in Golden Gate assembly has led to the development of standardized cloning systems with pre-defined fusion site sequences for different assembly fragments (e.g., promoters, terminators) [6–28]. These types of “modular” cloning systems allow labs to easily share assembly-ready fragments and have been developed for gene expression in bacteria, plants, and, eukaryotic cells.

Golden Gate assembly reactions have been successfully carried out with many different DNA ligase and Type IIS restriction enzyme combinations. Most assembly workflows utilize either T4 or T7 DNA ligase; T4 DNA ligase has been shown to be more efficient and less biased against A/T rich overhang sequences [29]. There are many commercially available Type IIS restriction enzymes, but most Golden Gate assembly workflows use BsaI, BsmBI, Esp3I, BbsI, SapI, or isoschizomers of these enzymes. Selection of a Type IIS restriction enzyme is typically guided by compatibility with a modular cloning system or a desired recognition sequence. Importantly, fragment sequences cannot contain additional Type IIS recognition sequences for the enzyme being used in the assembly, as the desired assembly product would be vulnerable to internal cleavage by the Type IIS restriction enzyme, preventing formation of full-length constructs. To circumvent this problem, users can choose a Type IIS restriction enzyme with a recognition sequence not present in the desired assembly sequence, or, alternatively, remove Type IIS recognition sequences from assembly fragments by mutagenesis [30,31]. BsaI, BbsI, and BsmBI (along with its isoschizomer Esp3I) each have a distinct six-base pair recognition sequence and cleave DNA to generate four-base overhangs with a 5′-phosphate. SapI is the most distinct of the commonly used Type IIS restriction enzymes, as it has a seven base pair recognition sequence and cleaves DNA to generate three-base overhangs with a 5′-phosphate [5]. The extended recognition sequence of SapI reduces the likelihood a desired target DNA sequence will contain an extraneous SapI recognition site, though permits fewer sequences at the fusion sites compared to those that generate four-base overhangs. It is currently unknown whether these commonly used Type IIS restriction enzymes exhibit a sequence bias at DNA cleavage sites under assembly conditions.

Selection of the overhang sequences that flank assembly fragments is an important consideration for successful Golden Gate assembly because promiscuous ligation of non-complementary overhang sequences by the DNA ligase can reduce assembly yield and increase the amount of time required to screen for the desired assembly product [29,31]. Typically, Golden Gate assembly reactions join 5–10 fragments per reaction, however, some modular cloning systems can accommodate up to 25 fragments in a single reaction [12]. The overhang sequences connecting fragments are selected using broad design guidelines that minimize base pairing between non-complementary overhangs. This includes avoiding use of palindromic overhang sequences or the same overhang pair more than once in an assembly reaction. In addition, most modular cloning systems also require that non-complementary overhang sequences have at least 2 mispaired bases. Moreover, overhangs that contain 100% A/T or G/C content are also often avoided as these overhangs are thought to join with low efficiency, and, sometimes, overhangs with 25% G/C content are also avoided for the same reason. These design principles can inconveniently limit the design of complex assemblies, as the ideal breakpoints in a DNA sequence of interest may call for violation of these rules. Furthermore, these guidelines are laborious to implement when designing assemblies by hand, especially for assemblies of >10 fragments.

Several recent reports have attempted to improve assembly design by utilizing experimental data to inform overhang selection. One recent study examined intra-molecular digestion and ligation of DNA substrates with T4 DNA ligase in conjunction with BsaI and used these data to provide recommendations for overhang sets anticipated to join with high efficiency and fidelity [31]. However, the scope of this study was limited to approximately 80% of the possible sequence contexts, and the use of only one Type IIS restriction enzyme. In a recently published report from our lab, we found DNA ligation fidelity could be used to estimate the fidelity of Golden Gate assembly reactions [29]. For example, we accurately predicted the fidelity of a 25-fragment assembly reaction with T4 DNA ligase and BsaI-HFv2, using data from ligation reactions with T4 DNA ligase alone. However, it is unclear if the data from this study is broadly applicable to designing assembly reactions with other Type IIS restriction enzymes. Finally, both studies were limited to four-base overhangs, and there are currently no resources available to guide assembly design with three-base overhangs, such as those generated by SapI.

To facilitate the design of large, multiple fragment assembly reactions, we systematically examined digestion and ligation of every overhang sequence combination under typical Golden Gate assembly reaction conditions using T4 DNA ligase and BsaI-HFv2, BsmBI-v2, Esp3I, BbsI-HF or SapI. We find the choice of a Type IIS restriction enzyme marginally impacts the observed assembly efficiencies for each overhang pair, suggesting that cleavage is robust with the commonly used Type IIS restriction enzymes under typical Golden Gate reaction conditions. We also note mispairing is common in assembly reactions, and the observed assembly outcomes are complex and not trivially reduced to simple trends or rules. Thus, the application of these data sets to design assembly reactions by hand would be difficult. To address this limitation, we developed a suite of user-friendly Golden Gate assembly webtools leveraging this data for computer-assisted Data-optimized Assembly Design (DAD). These tools enable users to check the estimated assembly fidelity of overhang sets, generate customized high-fidelity overhang sets, and divide a target sequence into high-fidelity assembly fragments. Using these tools, we demonstrate how to troubleshoot and expand sets of overhangs for modular cloning systems as well as estimate the limits of high-fidelity assembly. Lastly, we use DAD to design and carry out the most complex one-pot Golden Gate assembly reactions to date: 13-fragment assembly with three-base overhangs and 35-fragment assembly with four-base overhangs.

## Results

**Fig 1:**
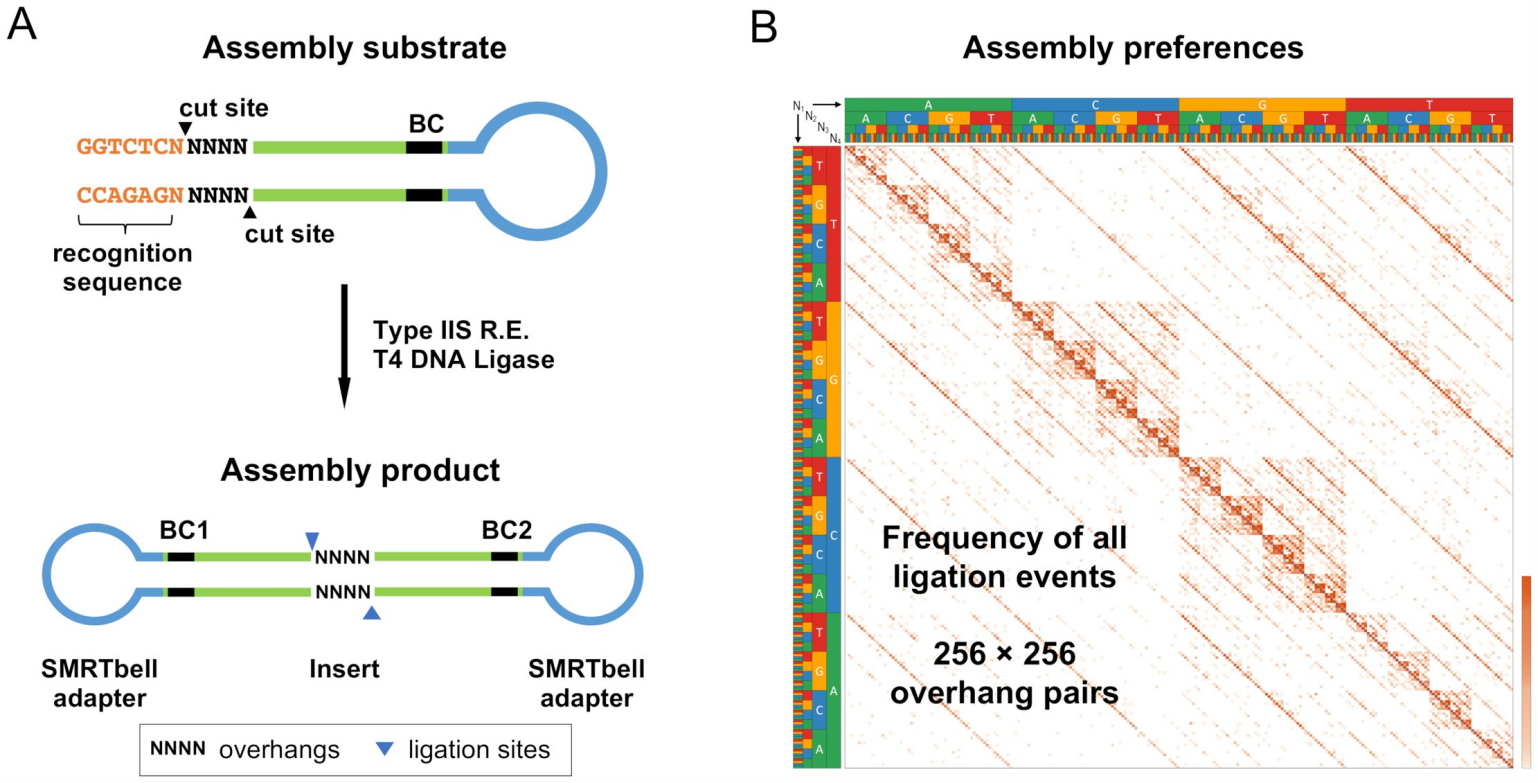
Golden Gate assembly assay schematic. (A) Hairpin DNA substrates containing a Type IIS recognition sequence (orange), randomized nucleotides at the Type IIS restriction site (NNNN), an internal 6-base random barcode (black), and a PacBio SMRTbell adapter sequence (blue) were synthesized. Golden Gate assembly of these substrates was carried out with T4 DNA ligase and a Type IIS restriction enzyme to produce circular assembly products. The assembly products were sequenced utilizing the PacBio Single-Molecule Real-Time sequencing platform. (B) For each sequenced assembly product, the overhang pair identity was extracted. The relative frequency of each overhang pair was determined and was represented as a frequency heat map (log-scaled). Overhangs are listed alphabetically left to right (AAAA, AAAC…TTTG, TTTT) and bottom to top such that the Watson–Crick pairings are shown on the diagonal represented above.

To profile the details of fidelity and bias in Golden Gate assembly reactions, we employed a modified version of our previously reported high-throughput, single-molecule DNA sequencing assay. This assay was initially designed to study DNA ligation [29,32]; the modified version was redesigned to more closely mimic the features and reaction conditions of a Golden Gate assembly reaction. Briefly, we generated hairpin DNA substrates containing Type IIS restriction enzyme recognition sites and Pacific Bioscience (PacBio) single-molecule, real-time sequencing (SMRT)-bell adapter sequences (Fig 1A). Importantly, each substrate also includes a segment of randomized bases at the Type IIS restriction site with equal representation of A, T, G, and C nucleotides at each position. This design enables examination of every overhang sequence context in the same assembly reaction. The hairpin substrates were combined with T4 DNA ligase and a Type IIS restriction enzyme and assembly reactions were carried out using a thermocycling protocol. The resulting assembly products were sequenced using the PacBio Single-Molecule Real-Time sequencing platform (Fig 1B). The relative frequency of each overhang pair indicates the relative efficiency with which each pair was joined. Using these data, we can estimate Golden Gate assembly fidelity by comparing the assembly efficiencies of Watson-Crick pairs to mispairs, and bias by examining the relative efficiency of each Watson-Crick pair.

**Fig 2:**
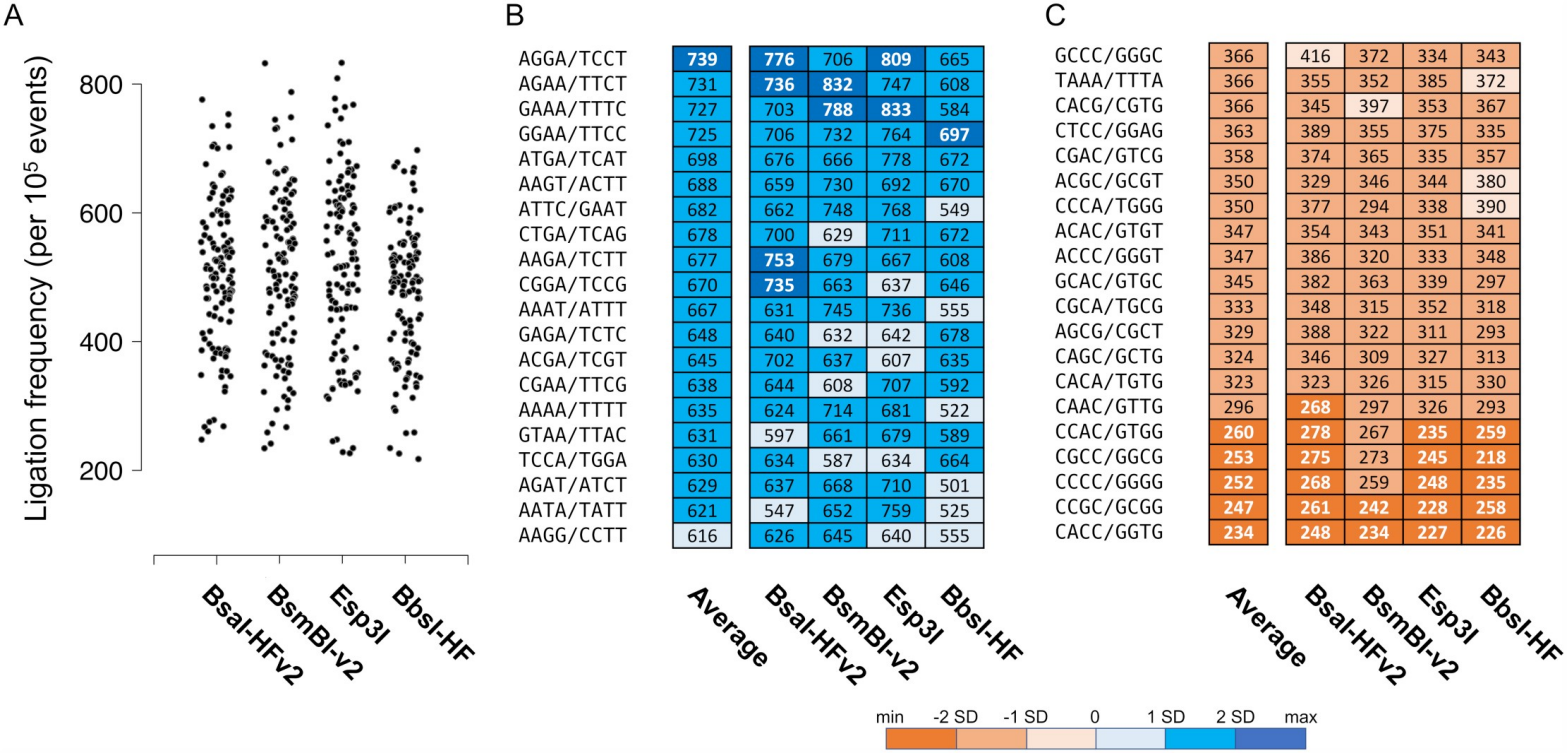
Assembly bias with T4 DNA ligase and Type IIS restriction enzymes generating four-base overhangs. (A) The normalized overhang ligation frequencies for all 120 non-palindromic Watson-Crick pairs were plotted for DNA assembly reactions containing T4 DNA ligase and the indicated Type IIS restriction enzyme. (B-C) The most and least frequently observed overhang pairs and their relative frequency per 100,000 ligation events are shown. The overhangs are written in a 5′ to 3′ orientation. The overhang pairs are color-coded according to their frequency relative to the average in terms of the number of standard deviations.

**Fig 3:**
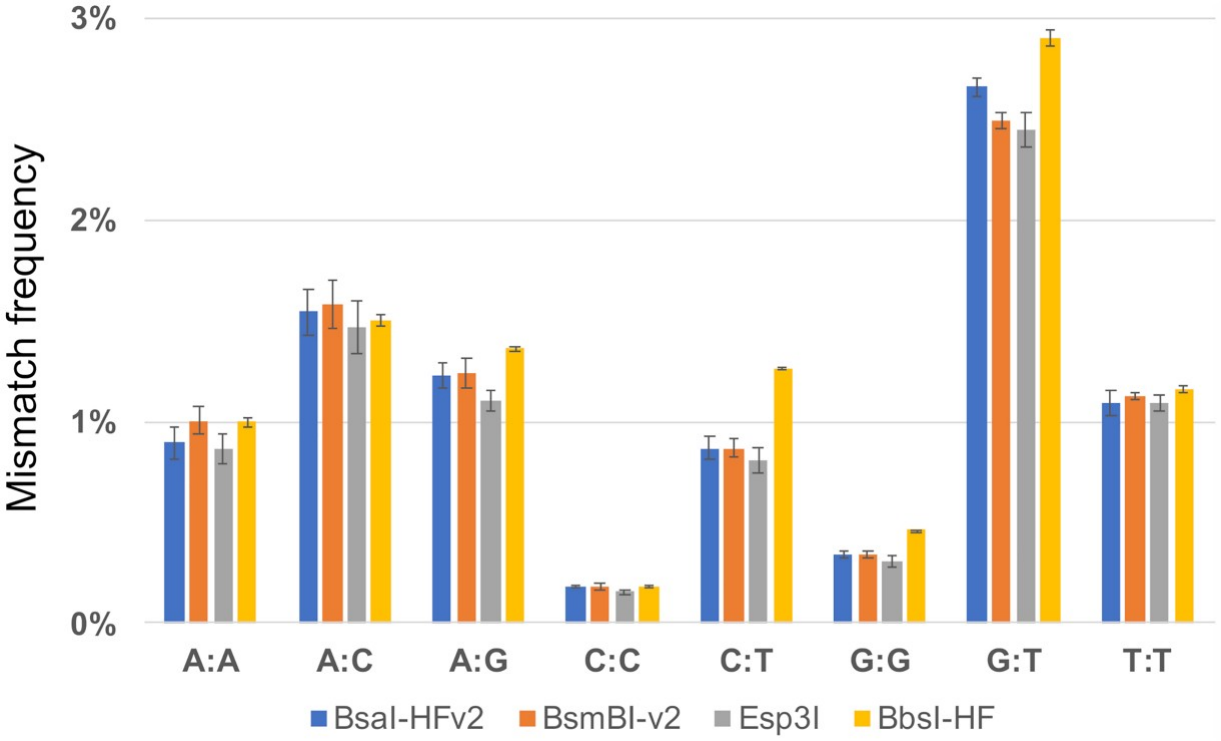
Nucleotide mismatches in assembly reactions with T4 DNA ligase and Type IIS restriction enzymes generating four-base overhangs. Mismatch frequencies for assembly reactions with T4 DNA ligase and BsaI-HFv2 (blue), BsmBI-v2 (orange), Esp3I (gray), or BbsI-HF (yellow) were grouped according to nucleotide mispair (A:A, A:C, A:G, C:C, C:T, G:G, G:T, T:T). The error bars depict the range between the maximum and minimum observed mismatch frequencies for two experimental replicates.

In our previous study of DNA ligation fidelity, we found T7 DNA ligase inefficiently ligates A/T-rich four-base overhang sequences [29]. To determine whether the choice of Type IIS restriction enzyme similarly introduces bias into Golden Gate assembly reactions, we first examined assembly with T4 DNA ligase and commonly used Type IIS restriction enzymes that generate four-base overhangs: BsaI-HFv2, BsmBI-v2, Esp3I, and BbsI-HF. In each of the assembly reactions we observed the presence of all 128 Watson-Crick overhang pairs and >2000 mismatch pairs (S1–S4 Tables). The range and distribution of assembly efficiencies for the Watson-Crick pairs were similar regardless of the restriction enzyme used (Fig 2A). In addition, the assembly efficiency of each Watson-Crick pair was well correlated (Fig 2B and 2C). Finally, the frequency of each nucleotide mispair was also similar among the assembly reactions and approximates the mismatch tendencies previously reported for T4 DNA ligase alone (Fig 3) [29]. Taken together, these data suggest that assembly fidelity and bias is not significantly impacted by choice of the Type IIS restriction enzymes and is instead determined primarily by the DNA ligase and reaction conditions, as previously proposed [29]. However, in comparison with our previous ligation fidelity study, we note higher frequencies of mismatch pairs and less bias against A/T-rich overhang sequences under Golden Gate assembly conditions. Presumably, this is due to differences in the reaction temperatures and buffer conditions between the two studies.

Given that the fidelity and bias were similar regardless of restriction enzyme used, we examined how the assembly data could be used to guide junction selection for Golden Gate assembly with four-base overhangs. Regarding the relative efficiencies of each overhang pair, we found that all the Watson-Crick pairs were assembled >10-fold more efficiently than the most efficiently joined mispairs (S1–S4 Tables). We also noted that the relative efficiency of each Watson-Crick pair was not simply a function of GC content, and thus, difficult to predict based on the sequence composition alone. For example, we found that assembly of the 5′-ATTT/5′-AAAT overhang pair is significantly more efficient than assembly of the 5′-TTTA/5′-TAAA pair. Similarly, the efficiency was not always well correlated with the identity of the nucleotide bases adjacent to the ligation site; for example, the 5′-TCCG/5′-CGGA pair is assembled significantly more efficiently than the 5′-TGTG/5′-CACA pair (Fig 2B and 2C). Regarding assembly fidelity, we found that each overhang typically mispaired with >15 non-complementary partners, but usually had only 2–3 efficient mispair partners (S1 Fig, S1–S4 Tables). These data suggest that none of the non-palindromic Watson-Crick overhang pairs are inherently low fidelity, as small sets comprising <5 overhangs would be expected to join with high-fidelity in almost every case. However, as the size of the overhang set increases mismatch ligation becomes more problematic. Taken together, these data further support and emphasize the difficulty of designing complex assemblies by hand.

To help guide overhang selection for assemblies with Type IIS restriction enzymes that generate three-base overhangs, we examined Golden Gate assembly with T4 DNA ligase and SapI. We observed all 32 Watson-Crick overhang pairs and >500 distinct mispairs in the assembly products (S5 Table). We again noted that assembly was promiscuous, as each overhang usually mispaired with >10 non-complementary partners (S1 Fig). In contrast to Golden Gate assembly with four-base overhangs, we found that non-palindromic self-mismatches were among the most frequently observed mismatch pairs (Table 1). For example, 5′-CTG/5′-CTG is a frequently observed mispair that significantly decreases the anticipated assembly fidelity for reactions using the 5′-CTG/5′-CAG Watson-Crick pair. This mismatch is likely due to ligation promiscuity when a T is flanked by a strong nucleotide pair, as 5′-CTC/5′-GTG is also a frequently observed mismatch pair. In addition, we again found that the prediction of efficient overhang combinations is non-trivial based on the sequence composition of the overhang. For example, 5′-AAT/5′-ATT is among the highest efficiency overhang pair, whereas 5′-TTA/5′-TAA is joined inefficiently (Table 1). Thus, we anticipate assembly design could be significantly improved by selection of overhangs on a case-by-case basis, rather than using broad guidelines for overhang sequence selection.

**Table 1:** Assembly bias with T4 DNA ligase and SapI.

Watson-Crick pair	Relative frequency ^a^	Mismatch pair^b^	Relative frequency ^a^
ATC/GAT	420	GCC/GGT	34
AAT/ATT	409	GCT/GGC	34
ACT/AGT	382	GAT/GTC	33
GAC/GTC	380	CTC/GTG	29
ATG/CAT	373	GAC/GTT	25
AGG/CCT	342	ACG/CGC	22
AAG/CTT	342	CGT/GCG	20
AAC/GTT	334	ACC/GGC	19
AGC/GCT	333	ACT/GGT	19
CTC/GAG	331	CTG/CTG ^**c**^	19
ATA/TAT	322	CCT/GGG	19
GTA/TAC	322	ATG/CTT	18
AGA/TCT	319	GGG/TCC	18
GCC/GGC	312	GCG/TGC	18
ACC/GGT	309	AGT/GCT	17
ACA/TGT	302	GTC/GTC ^**c**^	15
CAG/CTG	282	ACG/CGG	15
ACG/CGT	280	AGC/GCC	14
GCA/TGC	278	CAT/GTG	14
GGA/TCC	274	ACC/GGG	14
CCC/GGG	260	GCC/GTC	13
CTA/TAG	256	CTT/GAG	13
GAA/TTC	246	AAT/GTT	12
CAC/GTG	243	GGC/GTC	12
CGC/GCG	218	AAC/GTA	12
CCA/TGG	213	ATT/GAT	11
CAA/TTG	208	ACC/GGA	11
CCG/CGG	194	AAC/GTC	11
AAA/TTT	165	ATC/GTT	10
CGA/TCG	143	AGC/GCA	10
TCA/TGA	96	GCC/GGA	10
TAA/TTA	64	AGG/TCT	10

^a^ Ligation frequencies were normalized to 10,000 ligation events.

^b^ Thirty-two most common mismatch pairs were presented.

^c^ Overhangs with a non-palindromic self-mismatch.

### New tools enable Data-optimized Assembly Design (DAD)

To design Golden Gate assembly reactions using our sequencing data, we developed a suite of tools to parse the data in several different ways. The Ligase Fidelity Viewer is used to check assembly fidelity of overhang sets, the GetSet tool is used to design high-fidelity overhang sets, and the SplitSet tool is used to divide up a target DNA sequencing into high-fidelity assembly fragments. These tools, described in more detail below, are freely available on the web at: https://www.neb.com/research/nebeta-tools.

**Fig 4:**
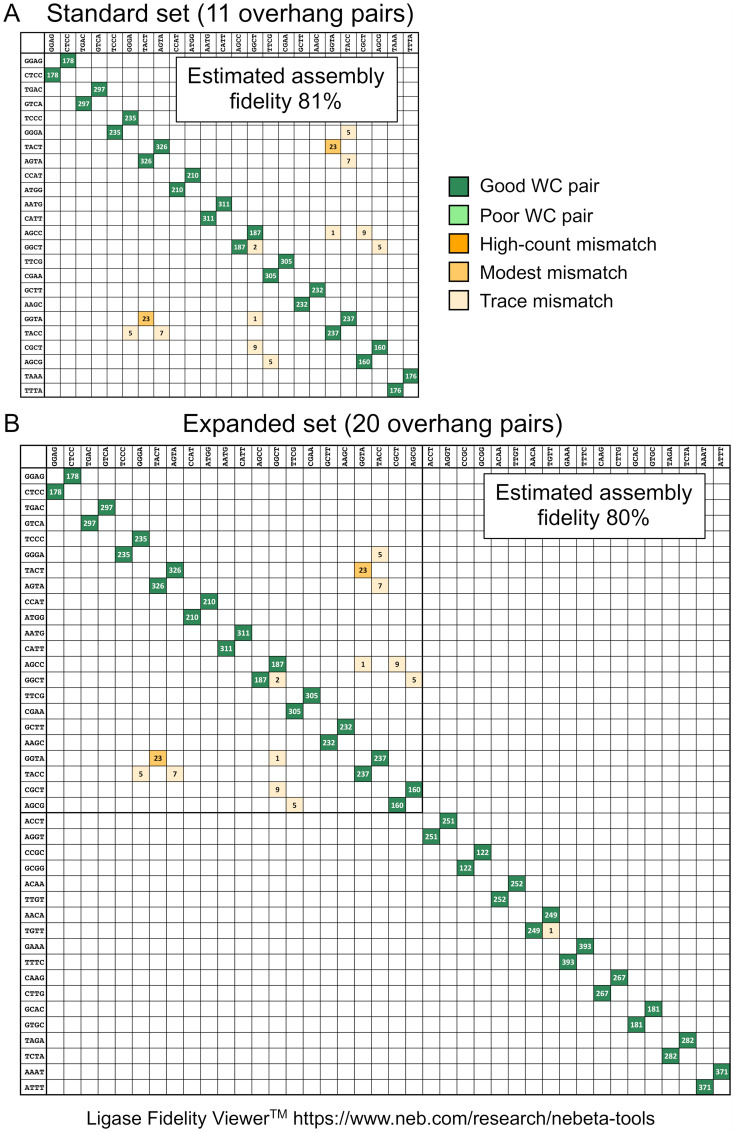
Example of data-optimized assembly design. (A) The Ligase Fidelity Viewer was used to estimate assembly fidelity of the 11 standard overhangs used in plant synthetic biology (GGAG, TGAC, TCCC, TACT, CCAT, AATG, AGCC, TTCG, GCTT, GGTA, CGCT). Overhang sequences are written 5′ to 3′. For this example, we choose the BsmBI-v2 restriction enzyme and 42°C/16°C thermocycling protocol. Under these conditions, the estimated assembly fidelity for this set was 81%. (B) The GetSet tool was used to add 9 additional overhangs (ACCT, CCGC, ACAA, AACA, GAAA, CAAG, GCAC, TAGA, AAAT). The estimated assembly fidelity for the combined set of 20 overhangs was 80%.

We developed the Ligase Fidelity Viewer to estimate assembly fidelity for a given set of user-supplied overhangs and identify problematic overhang pairings with a high potential for mismatch ligation. To use this tool, users input a set of three-base or four-base overhang sequences and select the desired Type IIS restriction enzyme and thermocycling protocol. The Ligase Fidelity Viewer then returns an estimated fidelity for assembly, along with an assembly matrix that identifies potential mismatch connections. As an example, we used the Ligase Fidelity Viewer to check the fidelity of an assembly that uses the standard overhang set for plant synthetic biology [33]. We found that 81% of the assembly products are predicted to be error-free when using this overhang set (Fig 4A). Notably, most of the assembly errors are expected to result from the 5′-GGTA/5′-TACT mispair, and avoiding this pair increases the predicted assembly fidelity to 92%.

The GetSet tool allows users to generate overhang sets with maximum assembly fidelity using automated overhang selection. To use this tool, users enter the desired overhang set size and overhang length (three-base or four-base), and GetSet returns a high-fidelity overhang set matching the input criteria. Users can specify overhang sequences that must be included or excluded from the results. Importantly, GetSet does not use pre-calculated results and instead identifies *de novo* high-fidelity overhang sets using a stochastic search algorithm. Consequently, the stochastic search algorithm may return different recommended overhang sets from the same input criteria, meaning repeating a search can result in different junction sets with similar predicted fidelities. We have therefore included a feature to save and recall prior GetSet search results. As an example, we used the GetSet tool to expand the standard overhang set used in plant synthetic biology; we found the set size could be increased from 11 overhangs to 20 overhangs with marginally decreasing the predicted assembly fidelity from 81% to 80% (Fig 4B).

While the GetSet tool is ideally suited for users wishing to design or expand sets of standardized overhang connection sequences that may be used regardless of the sequence of the DNA fragments, identifying high-fidelity breakpoints at convenient locations within a fixed sequence (e.g., coding sequence) could be difficult using this tool. Therefore, we designed the SplitSet tool to efficiently design high-fidelity assembly fragments from a desired target DNA sequence. To use this tool, users input a DNA sequence, the desired number of fragments, and approximate search windows for fusion sites (by default, the program chooses equally spaced search intervals). The SplitSet tool will then divide the input DNA sequence at the highest fidelity set of junctions within the parameters chosen. In addition, users can exclude specific fusion site sequences to ensure compatibility with pre-existing modular cloning systems or include fixed sites by setting a narrow search window to cover which site or sites must be used.

### DAD increases the fragment capacity of Golden Gate assembly

**Fig 5:**
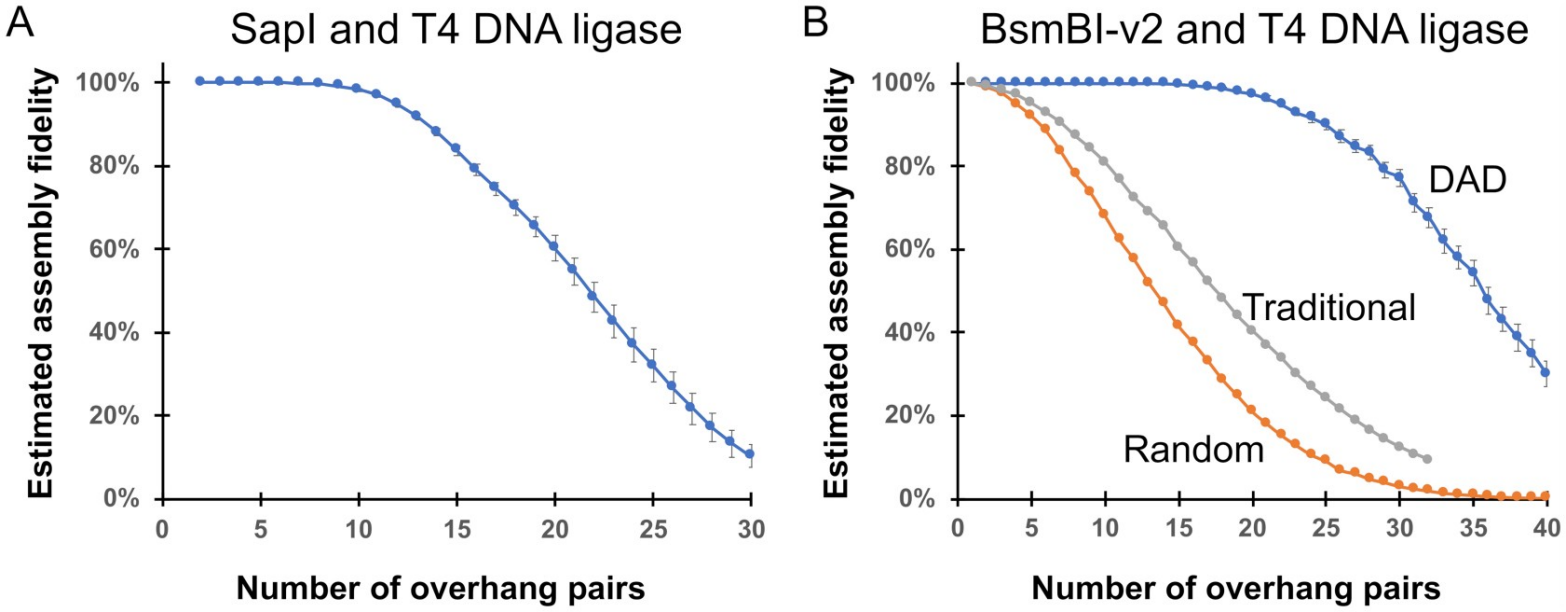
Golden Gate assembly fidelity predictions as a function of the overhang pairs in the assembly reaction. (A) The GetSet tool was used to estimate the fidelity of assembly reactions containing up to 30 overhang pairs with T4 DNA ligase and SapI. (B) GetSet was used to estimate assembly fidelity for overhangs sets with up to 40 overhang pairs in an assembly reaction with T4 DNA ligase and BsmBI-v2. Overhang pairs were selected using Data-optimized Assembly Design (DAD; blue), traditional rules for overhang selection by hand (gray), or by random overhang selection of non-palindromic overhang pairs (orange). The error bars indicate estimated fidelity scores based on replicate data analysis (see S1 Text for details).

Golden Gate assembly reactions utilizing Type IIS restriction enzymes that generate three-base overhangs are currently limited to approximately 5 fragments per assembly reaction [24,27]. We sought to determine if using DAD could significantly increase the fragment capacity of these cloning systems. To estimate how many fragments could be faithfully assembled using three-base overhangs, we used the GetSet tool to identify high-fidelity overhangs sets for assemblies with T4 DNA ligase and SapI (Fig 5A). The GetSet tool identified overhang sets containing up to 10 overhang pairs predicted to join >99% accurately, however the predicted fidelity for assemblies with sets containing 11–30 overhangs pairs decreased with each additional overhang added to the set. Assembly reactions utilizing Type IIS restriction enzyme generating four-base overhangs are typically limited to approximately 5–10 fragments per reaction [34], though several recent studies have demonstrated that it is possible to combine up to 25 fragments in one reaction [12,29]. To determine if DAD could be used to increase the capacity of these cloning systems, we repeated our overhang set analysis for assemblies with T4 DNA ligase and BsmBI-v2 (Fig 5B). The GetSet tool identified overhang sets predicted to join with perfect fidelity until the desired number of overhang pairs exceeded approximately 20, after which the predicted assembly fidelity decreased as the size of the overhang set increased. We repeated this analysis with T4 DNA ligase and BsaI-HFv2, Esp3I, or BbsI-HF, and did not observe significant differences between assembly reactions with the different Type IIS restriction enzymes (S3 Fig). Taken together, these data suggest that DAD could significantly increase the fragment capacity of Golden Gate assembly reactions.

During examination of the high-fidelity overhang sets generated by the GetSet tool, we noticed that many overhang sequences in these sets violate the traditional rules for designing overhang sets by hand. To compare assembly design by DAD with the traditional overhang design standards, we repeated our fidelity predictions for overhang sets generated using the traditional design rules. For comparison, we also analyzed the fidelity of overhang sets that were selected at random. Using the traditional overhang design standards, we could identify high-fidelity overhang sets containing approximately 10–12 overhang pairs (Fig 5B). Using randomly selected non-palindromic overhang pairs, we identified overhang sets containing up to 6–8 overhang pairs anticipated to join with high-fidelity. This analysis suggests the traditional overhang design rules offer a clear improvement over random overhang selection; however, DAD can be used to identify much larger sets of high-fidelity overhangs, with the added advantage of eliminating the laborious task of selecting overhangs by hand.

**Fig 6:**
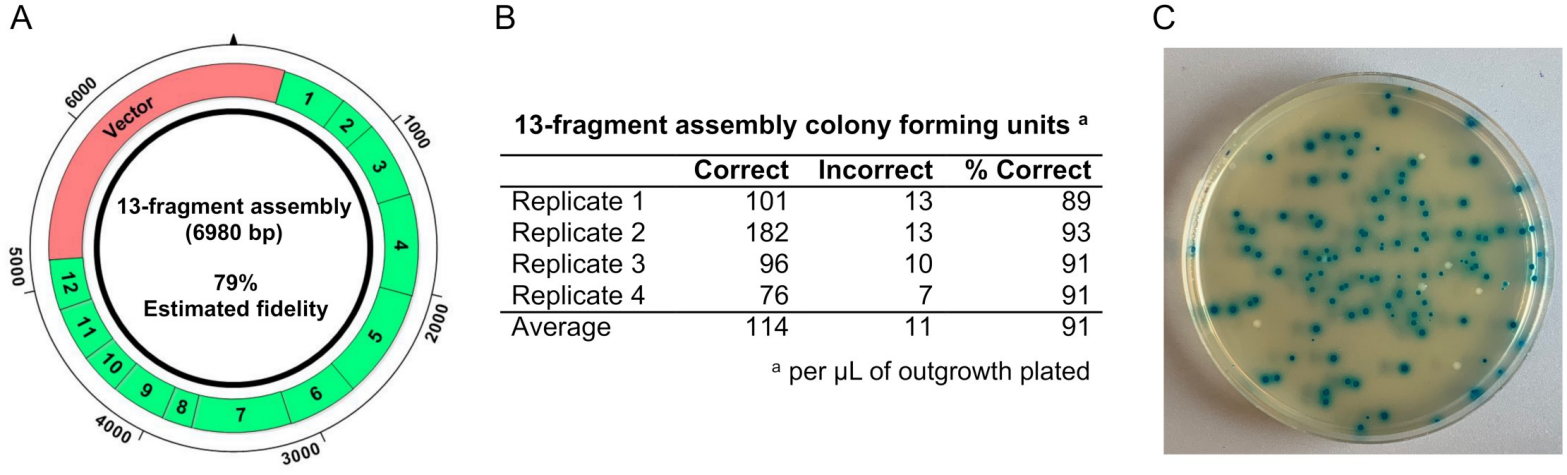
High capacity Golden Gate assembly with T4 DNA ligase and SapI. (A) Schematic of the 13-fragment *lac* operon cassette test system. (B) Results of the assembly reactions. Four replicate experiments were carried out to quantify the number of colony-forming units harboring correct and incorrect assembly products per μL of *E*. *coli* outgrowth plated (0.002 μL of the assembly reaction). On average, 91% of the observed transformants harbored correctly assembled products. (C) Representative agar plate with blue and white colonies. Blue transformants harbor correct assembly constructs, and white transformants harbor inaccurate assembly products.

To test the GetSet/SplitSet predictions in a practical application, we first designed a 13-fragment assembly test system using three-base overhangs, with an estimated assembly fidelity of 79% (Fig 6A, Table 2, and S6 Table). Assembly reactions were carried out with SapI and T4 DNA ligase, and the accuracy of assembly was assessed after transformation into *E*. *coli* cells using a reverse blue-white screen previously developed in the lab [29]. Briefly, the DNA assembly fragments comprise a cassette of the *lac* operon that is cloned into a destination vector containing an antibiotic resistance marker. Importantly, transformants harboring correctly assembled constructs turn blue after incubation on media containing IPTG and X-Gal, while transformants harboring constructs with assembly errors form white colonies. We found that on average 91% of the observed transformants were blue, indicating uptake of a correct assembly product (Fig 6B and 6C). This frequency was slightly higher than the predicted assembly fidelity of 79% and could reflect that some incorrect assembly products cannot be propagated in *E*. *coli* under antibiotic selection, such as those that fail to produce circular constructs. To ensure the observed transformants were the result of *in vitro* assembly and not assembly of the DNA fragments within the *E*. *coli* by cellular DNA repair mechanisms, we also carried out control reactions lacking SapI and T4 DNA ligase. Importantly, we did not observe any colonies upon transformation of these control reactions. To verify the blue colonies contained accurately assembled constructs, we subjected a subset of blue colonies to additional screening by colony PCR. We found that all the blue colonies subjected to additional screening harbored constructs of the expected size (S2 Fig). Taken together, these data verify the GetSet prediction that >10 fragments can be accurately assembled in a one-pot Golden Gate assembly reaction with SapI and T4 DNA ligase.

**Table 2:** Overhang pairs used in the 13-fragment and 35-fragment assembly reactions.

Type IIS restriction enzyme	Number of overhangs	Overhang sequences (written 5′ to 3′)	Estimated assembly fidelity
SapI	13	GGA, CTC, GTA, ATC, ACT, GCA, ACG, CTA, AGA, CCA, AAC, GAA, ATG	79%
BsmBI-v2	35	GGAG, CAGA, GGTA, AGTT, AATA, TAGG, CTTA, TGCG, TCAC, AGAA, ACGA, TCCA, ACAG, TAGC, ACCT, ATCA, CGAC, TTAT, TTTC, GAGT, GTTT, TCTT, AGCC, GACA, CTAA, TCAA, TACA, GCGG, ATGC, ATTG, GATG, GGGC, CGTT, CGGG, CCAT	65%

**Fig 7:**
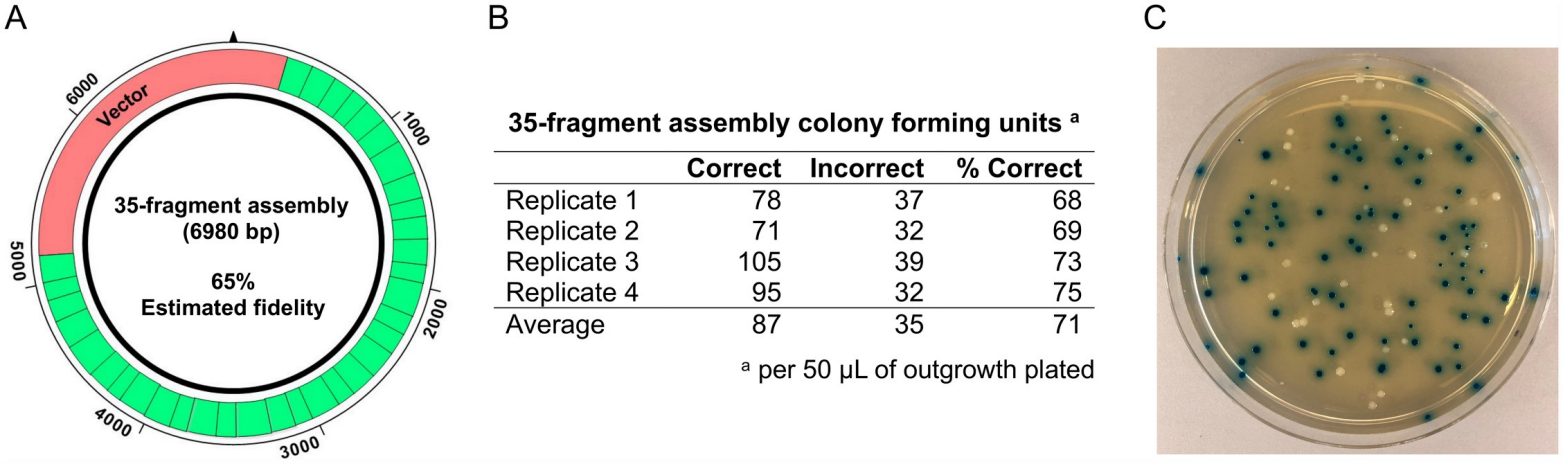
High capacity Golden Gate assembly with T4 DNA ligase and BsmBI-v2. (A) Schematic of the 35-fragment *lac* operon cassette test system (B) Results of the assembly reactions. Four replicate experiments were carried out to quantify the number of colony-forming units harboring correct and incorrect assembly products per 50 μL of *E*. *coli* outgrowth plated (0.1 μL of the assembly reaction). On average, 71% of the observed transformants harbored correctly assembled products. (C) Representative agar plate with blue and white colonies. Blue transformants harbor correct assembly constructs, and white transformants harbor inaccurate assembly products.

To test the fidelity predictions for assembly reactions with Type IIS restriction enzymes generating four-base overhangs, we designed a 35-fragment version of the *lac* operon cassette test system with a predicted assembly fidelity of 65% (Fig 7A and S7 Table). It should be noted the resulting overhang set contained several sequence combinations that are not allowed using traditional overhang design standards, including overhang pairs with 100% A/T or G/C content and many with only one base difference from multiple other members of the set (Table 2). We carried out assembly reactions using BsmBI-v2 and T4 DNA ligase and found that on average 71% of the observed transformants harbored accurately assembled constructs, compared to a theoretical prediction of 65% (Fig 7B and 7C). In addition, we noted assembly was robust, as we observed >700 transformants harboring correct assembly products per μL of the assembly reaction. Control reactions and additional screening on a subset of blue colonies were carried out as described above (S2 Fig). These data demonstrate that DAD can be used to easily design robust assembly reactions of unprecedented complexity, and we anticipate that utilizing these tools will be helpful to design robust assembly reactions of any size.

## Discussion

Here we provide a comprehensive analysis of Golden Gate assembly with T4 DNA ligase and a panel of commonly used Type IIS restriction enzymes. We found that the choice among commonly used Type IIS restriction enzymes that generate the same overhang structure did not considerably impact assembly fidelity and bias, suggesting that DNA cleavage is robust and not dependent on the restriction site sequence under standard Golden Gate assembly reaction conditions. These data support our previous work suggesting that Golden Gate assembly fidelity and bias is predominantly determined at the DNA ligation step [29,32]. Thus, the predicted fidelity of overhang sets is unlikely to be significantly impacted by the choice of Type IIS restriction enzyme, and this is likely broadly applicable to all Type IIS restriction enzymes that generate the same overhang structure including enzymes not explicitly tested here. However, it should be noted that we did not compare assembly yield between different Type IIS restriction enzymes. A more comprehensive study of reaction conditions involving thermocycling protocols, enzyme concentrations, and buffer conditions would be needed to compare the specific activity of enzyme mixes. Suboptimal conditions, such as temperature or buffer conditions where the activity of the restriction enzyme is poor and cutting is inefficient relative to re-ligation, could decrease the assembly yield. Thus, the choice of Type IIS restriction enzyme and the reaction conditions could significantly impact the assembly yield, and it is advisable to optimize the assembly reaction conditions, especially for assemblies with many fragments.

Selection of overhang standards for modular cloning systems has traditionally been labor intensive, but here we simplify the process by using bioinformatic tools to design overhang sets. Importantly, these tools can support the design of assembly reaction using Type IIS restriction enzymes that generate three-or four-base overhangs. We tested the predictions of these tools under challenging circumstances, to carry out very large multi-fragment assemblies, and found that the predictions closely matched the observed assembly fidelities. As noted in the results section, sets of overhangs that yield high-fidelity assemblies can contain individual overhangs which violate traditional overhang design rules. Thus, using comprehensive data sets to calculate predicted assembly fidelity and select fusion sites demonstrably leads to efficient assembly with few erroneous products, even when assembly complexity is much greater than current typical one-pot assemblies.

Importantly, predicted assembly fidelity should be taken as a qualitative prediction, most useful for comparing expected performance between alternative junction sets. In addition to uncertainty in the data used to estimate fidelity, other experimental factors such as suboptimal enzyme concentrations, thermocycling conditions, or DNA purity can influence both yield of the final assembly and prevalence of colonies lacking an insert or containing an undesired assembly. For example, DNA stock solutions contaminated with genomic DNA, a common source of contamination for DNA propagated in *E*. *coli*, can result in a high frequency of inaccurate assembly products due to inadvertent ligation of genomic DNA fragments into assembly products. Further, inaccurately quantified assembly fragments can substantially reduce assembly yield by favoring partially assembled constructs as limiting fragments are depleted. Moreover, impure DNA stock solutions may result in carryover contaminants, typically high concentrations of salt that can inhibit the enzymes in the golden gate reaction, reducing the yield of full-length constructs and/or leaving a large amount of uncut starting vector. Thus, we recommend DNA stock solutions be purified and accurately quantified for all assembly reactions to maximize assembly efficiency, in addition to selecting junction sets designed to minimize erroneous ligation events.

It is tempting to speculate that excluding Watson-Crick overhang pairs identified as low efficiency in our sequencing assay could likewise provide added benefit for assembly reactions. However, many experimental factors are expected to impact assembly efficiency, as described above. Additionally, we note the assembly efficiencies reported here are for assembly in the presence of every possible overhang combination and may underestimate the relative efficiency of the overhangs individually. This is especially true for overhang sequences that are prone to mismatch pairing. Practically, it should be noted that both the 13-fragment and 35-fragment assembly reactions contained many overhang pairs anticipated to join with relatively low efficiency, and we still obtained an ample number of transformants for both reactions. Thus, while it remains to be determined whether selecting only the highest efficiency pairs would enhance the assembly yield or decrease the time necessary to complete the reaction, it does not seem to be a major factor compared to DNA quality or assembly fidelity. Therefore, we suggest that assembly fidelity should be the primary consideration for the selection of overhang sets, and our tools are by default configured to select the highest fidelity overhang set that matches user specifications.

Here, we report the deployment of the SplitSet tool, which allows users to divide up a sequence into high-fidelity assembly fragments. In addition to allowing users to assemble large protein coding sequences or operons, we also anticipate that this tool could be utilized to quickly generate variants of assembly parts. Several recent studies have shown that placing assembly fusion sites close to mutational hot spots is a quick way to generate assembly ready amplicons containing sequence variations, as users can simultaneously carry out fragment amplification and PCR mutagenesis of the desired assembly fragment [30–31,35]. For example, this strategy could be used to easily remove internal Type IIS recognition sequences from assembly fragments, or easily generate high diversity libraries with randomized regions at specific sites, simply by setting windows for the junction fusion sites near the areas to be mutagenized.

As the field of synthetic biology continues to grow, rapid and robust build phases driven by highly efficient DNA assembly techniques are ever more critical. Golden Gate assembly has been particularly useful in many synthetic biology applications as it allows users to quickly generate construct variations with little *ad hoc* design from libraries of predefined DNA fragments. Here we provide webtools to guide assembly design and enable this technique to be implemented to its full potential, permitting unprecedented numbers of fragments to be assembled in a single step, and to ensure accurate assembly with less guesswork and less dependence on pre-vetted junction sets. These tools can be found at the following link: https://www.neb.com/research/nebeta-tools, and have also been integrated into a large suite of assembly webtools available at: https://goldengate.neb.com.

## Materials and methods

All enzymes, buffers, and media were obtained from New England Biolabs (NEB) unless otherwise noted. CutSmart® Buffer (1X) is: 20 mM Tris-acetate (pH 7.9), 50 mM Potassium Acetate, 10 mM Magnesium Acetate, 100 μg/ml BSA. T4 DNA ligase reaction buffer (1X) is: 50 mM Tris–HCl (pH 7.5), 10 mM MgCl_2_, 1 mM ATP, 10 mM DTT. NEBuffer™ 2 (1X) is: 10 mM Tris–HCl (pH 7.9), 50 mM NaCl, 10 mM MgCl_2_, 1 mM DTT. Standard Taq polymerase buffer is: 10 mM Tris-HCl (pH 8.3), 50 mM KCl, 1.5 mM MgCl_2_. Chemically competent *E*. *coli* strain T7 Express (NEB) lacks a functional lacZ gene, full genotype: fhuA2 lacZ::T7 gene1 [lon] ompT gal sulA11 R(mcr-73::miniTn10—TetS)2 [dcm] R(zgb-210::Tn10—TetS) endA1 Δ(mcrC-mrr)114::IS10. Column cleanup of oligonucleotides and ligated libraries was performed using Monarch PCR & DNA Cleanup Kit columns (NEB), following standard protocols. Oligonucleotide substrates were quantified by Agilent Bioanalyzer 2100, using a DNA 1000 assay, following the standard protocol. Synthetic oligonucleotides were obtained from either Integrated DNA Technologies (IDT) or Sigma Aldrich (Sigma).

### Golden Gate assembly fidelity and bias assay

DNA substrates for the sequencing assay were prepared as previously described [29, 32]. Briefly, cartridge-purified substrate precursor oligonucleotides were obtained as a lyophilized solid (Sigma). The substrate sequences (S8 Table) include: a 5′ Type IIS recognition sequence, a randomized four-base region, a constant region, an internal six-base randomized region as a control for synthesis bias, and a region corresponding to the SMRTbell sequencing adapter for PacBio sequencing. Each substrate precursor oligonucleotide (20 μM final concentration) was combined with 100 U of Klenow Fragment (3′→5′ exo^-^), 0.2 U yeast inorganic pyrophosphatase, 1 mM of each dNTP (final concentration), and 1X NEBuffer 2 reaction buffer (final concentration) in a 100 μl reaction volume. Extension reactions to generate full-length DNA substrates were carried out by incubation for 1 h at 37°C. Reactions were stopped by addition of 25 mM EDTA. The DNA was purified (Monarch PCR & DNA Cleanup Kit) and the concentration was determined using the Agilent Bioanalyzer 2100.

Reactions (20 μL final volume) with T4 DNA ligase and BsaIHF-v2 or BsmBI-v2 were carried out using their respective NEB Golden Gate Enzyme Mixes (2 μL) in 1X T4 DNA ligase buffer. The reactions (20 μL final volume) with T4 DNA ligase (500 U) and SapI (15 U) or Esp3I (15 U) were carried out in 1X T4 DNA ligase buffer. Reactions (20 μL final volume) containing T4 DNA ligase (500 U) and BbsI-HF (15 U) were carried out in 1X CutSmart Buffer supplemented with 10 mM DTT and 1 mM ATP. The final concentration of DNA substrate in each assembly reaction was 100 nM. The reactions were cycled between 37°C and 16°C (SapI, Esp3I, BsaI-HFv2, BbsI-HF) or 42°C and 16°C (BsmBI-v2) for 5 minutes at each temperature for 30 cycles, and then subjected to a final heat-soak for 5 minutes at 60°C. Reactions were then quenched by the addition of 25 mM EDTA and purified using the Monarch PCR & DNA Cleanup Kit. Each assembly reaction was performed a minimum of two times on different days. The assembly reactions were further purified to remove un-ligated substrates by treatment with Exonuclease III (50U) and Exonuclease VII (5 U) in 1X Standard Taq Polymerase buffer (final concentration) for 1 h at 37°C in a 50 μL reaction volume. The assembly products were then re-purified using the Monarch PCR & DNA Cleanup Kit, including a second wash step, and quantified by Agilent Bioanalyzer (DNA 1000).

PacBio Single-Molecule Real-Time sequencing was performed and the data analyzed as previously described [29,32]. Libraries were prepared for sequencing according to the PacBio Binding Calculator Version 2.3.1.1 and the DNA/Polymerase Binding Kit P6 v2 using the standard protocol, no-DNA control complex, and a custom concentration on plate (0.3375 nM). Libraries were sequenced on a PacBio RSII instrument with at least 2 SMRT cells per library and a 3 h data collection time per cell with ‘stage start’ off. Consensus sequences for each assembly product were generated as described previously [32]. Full results from each experiment are supplied in the supporting data files (S1–S5 Tables).

### Assembly tools development

Fidelity *F* for a set of *n* overhangs *{O*_*1*_, *O*_*2*_, *O*_*3*_, *…*, *O*_*n*_*}*, was defined as a probability that all overhangs in the set ligate correctly to their WC pair. Fidelity estimates the fraction of correctly ligated products when using a given set of overhangs in Golden Gate assembly and was computed as follows:

$$F=p\left(O_{1}\right)\times p\left(O_{2}\right)\times p\left(O_{3}\right)\times p\left(O_{n}\right),$$ (1)

where *p(O*_*i*_*)* is the probability of overhang *O*_*i*_ ligating correctly to its WC pair in a given set of overhangs. The probability *p(O*_*i*_*)* can be computed based on the observed number of ligation events in the experimental assembly data as follows:

$$p\left(O_{i}\right)=\frac{N_{correct}}{N_{total}},$$ (2)

where *N*_*correct*_ is the number of times overhang *O*_*i*_ ligates correctly to its WC pair and vice versa, and *N*_*total*_ is the number of times *O*_*i*_ ligates to any overhangs in the set and its WC pair. Therefore, for any set of overhangs, the fidelity can be easily estimated based on the observed number of ligations events.

To identify the highest fidelity set, it is necessary to find a combination of overhangs that maximizes the computed fidelity value (Eqs 1 and 2). For large sets, the number of possible combinations can be very large and exhaustive evaluation of all sets is computationally demanding. For four-base overhangs, there are 120 distinct overhangs after eliminating complementary and palindromic overhangs. The total number of combinations in such case is given according to the binomial coefficient $\left(\begin{array}{c} n\\ k \end{array}\right)$, where *n* is the set size, and *k* is the number of distinct elements, and exceeds 10^14^ combinations for 10-overhang sets. Instead, a stochastic Markov Chain Monte Carlo (MCMC) optimization technique was used to identify nearly optimal high-fidelity sets. Initially, a random set of *n* overhangs is generated, and its fidelity is estimated (*s*_*o*_). Then, a randomly chosen overhang in the set is replaced with another randomly chosen overhang and the fidelity for the new combination is estimated (*s*). If the new combination of overhangs improves the computed fidelity score (*s* > *s*_*0*_), it is accepted and used as a starting combination in the new iteration; otherwise (*s* < *s*_*0*_), the new combination is accepted according to the acceptance probability $p=e^{\left({s-s}_{0}\right)/T}$, where *T* is the temperature. The temperature *T* is an artificial parameter in our simulations that dictates how many unfavorable (*s* < *s*_*0*_) moves are accepted. A small number of initial random moves was conducted for each simulation to determine *T* at which, on average, 5% of unfavorable moves are accepted to avoid getting stuck in local optima. Subsequently, a larger number of iterations (1 × 10^4^) was performed at a given *T* and the best-found solution was reported. Additionally, a simulation with the linear annealing schedule was explored in which the temperature was varied in such a way, so that the acceptance ratio ranged from 95% to 0% throughout the course of simulation. We found that results were similar in both cases, however, the simpler implementation required less iterations to arrive at the similar optimum. It should be noted that other simulation annealing schedules can be used, however, the current optimization strategies already demonstrate an efficient convergence to the near optimum solutions.

#### Golden Gate assembly of the *lac* cassette test systems

Assembly fragments for *lac* cassette test systems were generated by PCR using Q5 DNA polymerase (2X hot-start master mix) with oligonucleotide primers (IDT). The sequences of each assembly fragment are provided in S6 and S7 Tables. Assembly fragments were purified using the Monarch DNA Cleanup Kit using a 1:1 ratio of sample: binding buffer, and the concentration was determined using the Agilent Bioanalyzer 2100.

Golden Gate assembly reactions (20 μL final volume) with SapI (15 U) and T4 DNA ligase (500 U) were carried out with 3 nM of each PCR assembly fragment in 1X T4 DNA ligase buffer. Reactions were cycled between 37°C and 16°C for 5 minutes at each temperature for 30 cycles, and then subjected to a heat-soak at 60°C for 5 minutes before being incubated at 4°C prior to transformation. Assembly reactions (20 μL final volume) with BsmBI-v2 and T4 DNA ligase (NEB Golden Gate assembly kit BsmBI-v2) were carried out with 3 nM of each PCR assembly fragment, 75 ng of pGGAselect destination vector, and 2 μL of NEB Golden Gate Enzyme Mix in 1X T4 DNA ligase buffer (final concentration). These reactions were cycled between 42°C and 16°C for 5 minutes at each temperature for 30 cycles, and then subjected to a 60°C incubation for 5 minutes and finally a 4°C hold until transformation.

All assembly products were transformed into T7 Express chemically competent *E*. *coli* cells, and the assembly fidelity was scored as described previously [29]. Briefly, transformations were performed using 2 μL of each assembly reaction added to 50 μL of competent T7 Express cells. Transformation reactions were incubated on ice for 30 min, and then incubated at 42°C for 10 s, with a final 5 minute recovery period on ice. SOC outgrowth medium (950 μL) was added and the cells were incubated 1 h at 37°C with vigorous rotation. The outgrowth was spread onto prewarmed agar plates (Luria–Bertani broth supplemented with 1 mg/mL dextrose, 1 mg/mL MgCl_2_, 30 μg/mL Chloramphenicol, 200 μM IPTG and 80 μg/mL X-gal). Plates were inverted and placed at 37°C for 18 h and then stored at 4°C for 8 h before scoring colony color phenotype.

## Supporting information

S1 Table Ligation frequency for each overhang pair in assembly reactions with BsaI-HFv2 and T4 DNA ligase. (XLSX)

S2 Table Ligation frequency for each overhang pair in assembly reactions with BsmBI-v2 and T4 DNA ligase. (XLSX)

S3 Table Ligation frequency for each overhang pair in assembly reactions with Esp3I and T4 DNA ligase. (XLSX)

S4 Table Ligation frequency for each overhang pair in assembly reactions with BbsI-HF and T4 DNA ligase. (XLSX)

S5 Table Ligation frequency for each overhang pair in assembly reactions with SapI and T4 DNA ligase. (XLSX)

S6 Table Fragment sequences for the 13-fragment *lac* operon cassette test system. (XLSX)

S7 Table Fragment sequences for the 35-fragment *lac* operon cassette test system. (XLSX)

S8 Table Sequences of the DNA substrate precursor oligonucleotides. (XLSX)

S1 Fig Mispair partners for each overhang sequence. Each datapoint represents a single overhang sequence. The four-base overhang data represents the average number of mispair partners for each overhang in assemblies with T4 DNA ligase and BsaI-HFv2, BsmBI-v2, Esp3I, and BbsI-HF; the three-base overhang data is the number of mispair partners observed in assembly reactions with T4 DNA ligase and SapI.
(TIF)

S2 Fig Colony PCR reactions. Blue colonies from both the 13-fragment (SapI + T4 DNA Ligase) and 35-fragment (BsmBI-v2 + T4 DNA Ligase) assembly reactions were subjected to PCR with amplification primers that flank the desired insertion site. We found that every blue colony produced an amplification product of the expected size for the accurate assembly product, demonstrating that blue colonies contained the desired number of inserts.
(TIF)

S3 Fig Estimated assembly fidelity for Golden Gate assembly with T4 DNA ligase and Type IIS restriction enzymes generating four-base overhangs. The GetSet tool was used to carry out data-optimized assembly design of reactions containing T4 DNA ligase and BsaI-HFv2, BsmBI-v2, Esp3I, or BbsI-HF. The number of overhang pairs in each assembly reaction was varied from 1 to 40.
(TIF)

S1 Text Error propagation analysis. (PDF)
