# Supplementary figures and images for: Enabling one-pot Golden Gate assemblies of unprecedented complexity using data-optimized assembly design
Source: PLoS One. 2020 Sep 2;15(9):e0238592. doi: 10.1371/journal.pone.0238592 (PMC7467295; doi:10.1371/journal.pone.0238592)

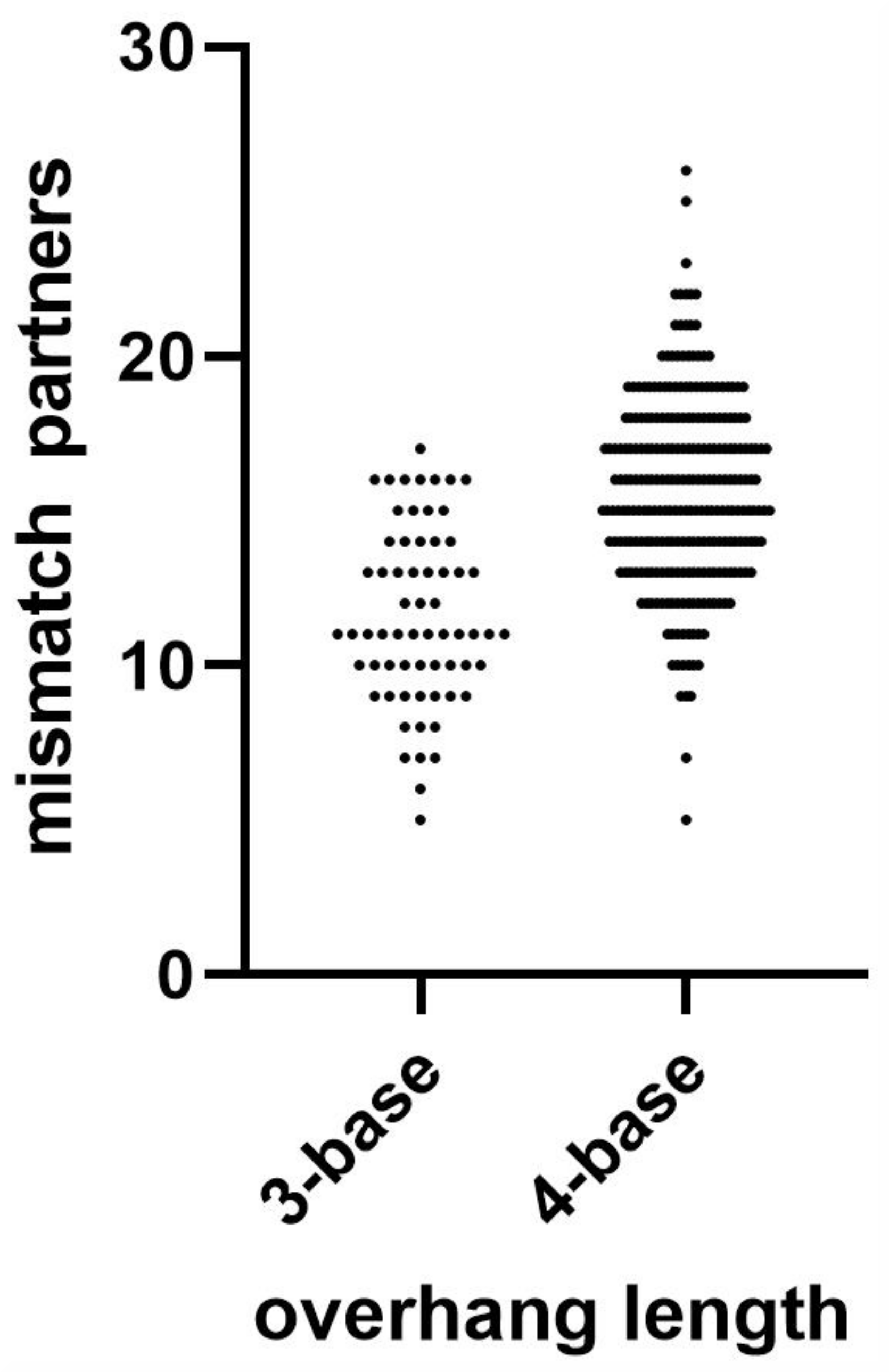

Supplement: S1 Fig — Each datapoint represents a single overhang sequence. The four-base overhang data represents the average number of mispair partners for each overhang in assemblies with T4 DNA ligase and BsaI-HFv2, BsmBI-v2, Esp3I, and BbsI-HF; the three-base overhang data is the number of mispair partners observed in assembly reactions with T4 DNA ligase and SapI. (TIF) [file pone.0238592.s009.tif]

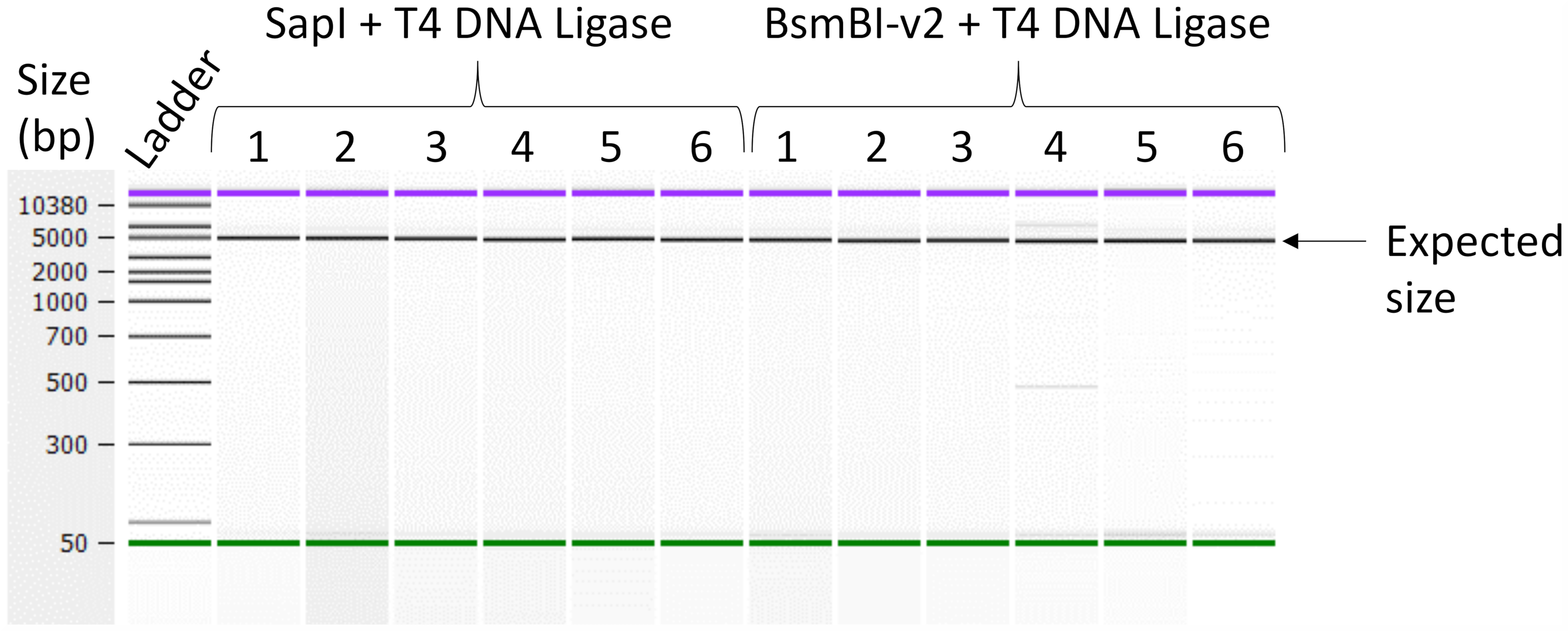

Supplement: S2 Fig — Blue colonies from both the 13-fragment (SapI + T4 DNA Ligase) and 35-fragment (BsmBI-v2 + T4 DNA Ligase) assembly reactions were subjected to PCR with amplification primers that flank the desired insertion site. We found that every blue colony produced an amplification product of the expected size for the accurate assembly product, demonstrating that blue colonies contained the desired number of inserts. (TIF) [file pone.0238592.s010.tif]

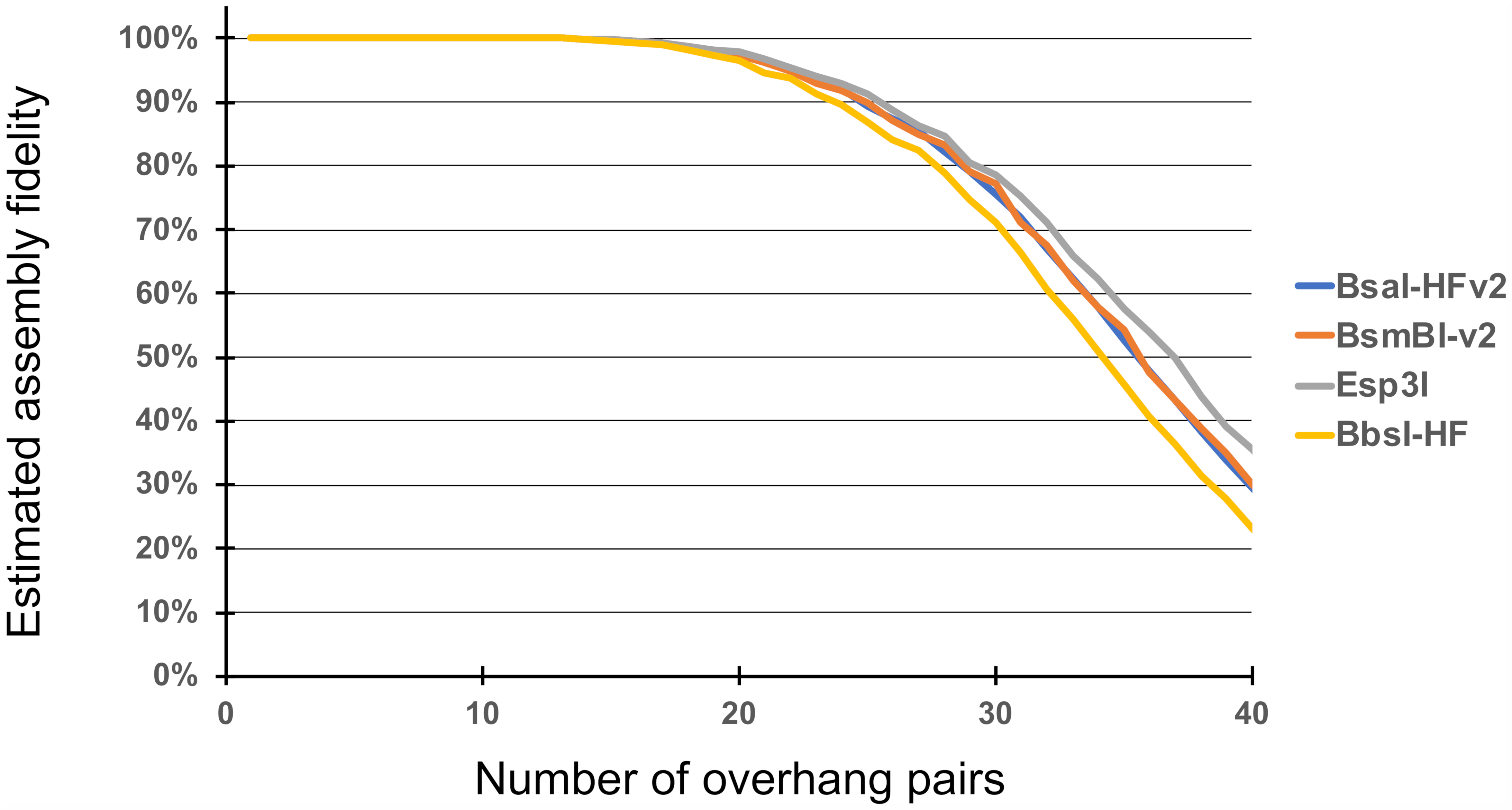

Supplement: S3 Fig — The GetSet tool was used to carry out data-optimized assembly design of reactions containing T4 DNA ligase and BsaI-HFv2, BsmBI-v2, Esp3I, or BbsI-HF. The number of overhang pairs in each assembly reaction was varied from 1 to 40. (TIF) [file pone.0238592.s011.tif]
