# Supplementary material for: Enabling one-pot Golden Gate assemblies of unprecedented complexity using data-optimized assembly design
Source: PLoS One. 2020 Sep 2;15(9):e0238592. doi: 10.1371/journal.pone.0238592 (PMC7467295; doi:10.1371/journal.pone.0238592)
Supplement: S1 Text — (PDF) [file pone.0238592.s012.pdf]

S1 Text. Error propagation analysis. As detailed in Materials and Methods, overhang set fidelity is calculated according to the equation Eq. 1 as follows: $F = p(O_1) \times p(O_2) \times p(O_3) \times \ldots \times p(O_n)$. It should be noted that there might be an inherent error in estimating $p(O_i)$, which are calculated based on the number of ligation events in our multiplexed Golden Gate Assembly assay according to Eq. 2. Theoretically, these errors might accumulate especially in situations with large numbers of fragments. To estimate the practical impact of variability in $p(O_i)$ on the computed fidelity score, independent replicate data from the multiplexed Golden Gate Assembly assay were used to score overhang sets. To this end, a large forty-overhang set was designed with the overall computed fidelity score of 41% based on combined BsmBI-v2 data (109,689 ligation events, in total): TCAA, ATAA, TAGA, GTTA, AGGG, CCTA, AAGA, TCCA, AGAA, AAAG, ACAT, GGGA, GCAA, TAAA, TGAA, GACA, ACGA, CCGC, GGTA, ATCC, ATTG, CTAC, AAGC, CATC, ACTC, CACA, CTAA, GAAA, AGAC, AGCA, CGCC, CGTA, ACCG, CAGA, AACT, AATA, GCAC, CCAG, CAAG, AAAT. All intermediate incremental sets ranging one to forty overhangs ({TCAA}, {TCAA,ATAA}, {TCAA,ATAA,TAGA}, etc.) were also scored and their computed fidelities were plotted in S4 Fig (black line). The same incremental overhangs sets were scored separately using BsmBI data obtained from independent replicate 1 (52,896 ligation events, in total) and replicate 2 (56,793 ligation events, in total). The fidelity scores computed based on independent replicates (light green and light blue lines) were similar to each other and to the computed fidelity scores based on the combined data (black line) despite the two-fold difference in the amount of data. However, it is evident that the range of predicted fidelity scores does increase with the number of overhangs in the set. Additionally, as the worst-case scenario, for each of the forty overhangs in the set the minimum and the maximum probability was picked out of two replicate values. The incremental sets were then scored using either all minimum probabilities or all maximum probabilities (S4 Fig; dark green and dark blue line for the minimum and maximum curve, respectively).

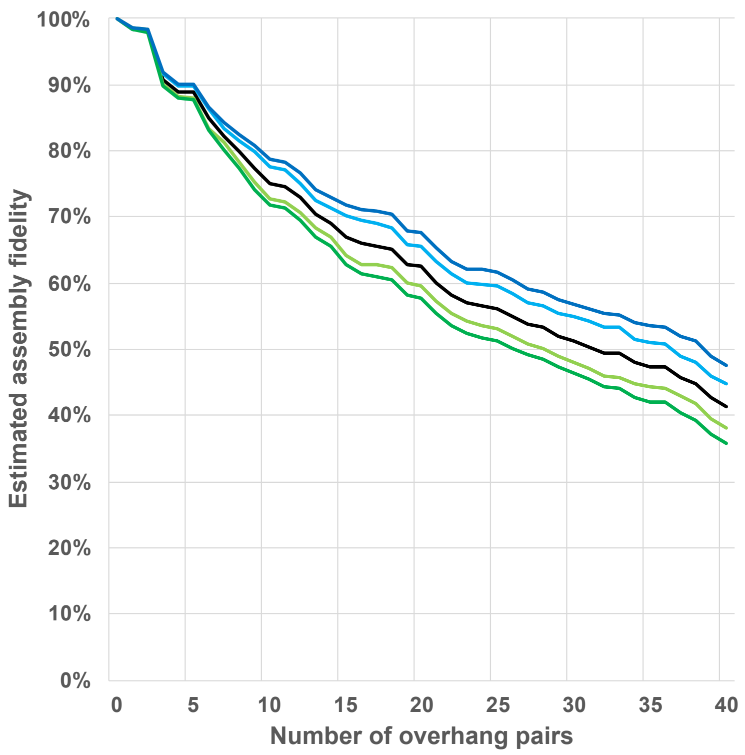

S4 Fig. Error propagation analysis for BsmBI-v2 computed fidelity scores on the forty-overhang set. Fidelity scores were computed incrementally for a forty-overhang set using combined BsmBI-v2 data, replicate 1 and replicate 2 data (black, light green and light blue lines, respectively). The minimum and maximum curves based on replicate 1 and 2 are shown in dark green and dark blue, respectively. See Supporting Information for further details.

The resulting fidelity score for the entire forty-overhang set was 41% with variation from 38% to 45% observed based on replicate data. The same analysis was conducted for BsaI-HFv2, Esp3I, and BbsI-HF multiplexed Golden Gate Assembly experiments, and the observed fidelity scores and their variation were 38% (35-40%), 44% (39%-50%), and 28% (24%-30%), respectively. This is well within variation one might expect from day-to-day experimental variations alone.

The observed error propagation for the computed fidelity scores is dictated by the agreement in the relative number of ligation events in replicate experiments and by the derived $p(O_i)$ for a particular overhang set. In fact, for the forty-overhang set, individual $p(O_i)$ were computed based on the independent replicates, and the mean difference was 0.7% for BsmBI experiments (1%, 0.9%, and 1.2% for BsaI-HVv2, Esp3I, and BbsI-HF, respectively). Therefore, even independent

replicates provide reproducible relative ligation counts and result in consistent computed fidelity scores. For computing the regular fidelity scores, the combined data is used which should provide even more reliable estimates. It should be noted that in each of our multiplexed Golden Gate Assembly assay a large number of ligation events are sampled, with each Watson-Crick pair sampled close to 500 times, on average.

In summary, the error might propagate for the computed fidelity scores; however, in the observed replicate data this leads to ~7% variation, on average, for a forty-overhang set. Sufficient sampling of ligation events in the multiplexed golden Gate Assembly assays is required to minimize such propagation for the computed fidelity scores.
